# Synopsis of *Leptosphaeriaceae* and Introduction of Three New Taxa and One New Record from China

**DOI:** 10.3390/jof8050416

**Published:** 2022-04-19

**Authors:** Rong Xu, Wenxin Su, Shangqing Tian, Chitrabhanu S. Bhunjun, Saowaluck Tibpromma, Kevin D. Hyde, Yu Li, Chayanard Phukhamsakda

**Affiliations:** 1 Internationally Cooperative Research Center of China for New Germplasm Breeding of Edible Mushroom, Jilin Agricultural University, Changchun 130118, China; xurong@jlau.edu.cn (R.X.); wenxinsu@yahoo.com (W.S.); 13689830727@163.com (S.T.); 2 College of Plant Protection, Jilin Agricultural University, Changchun 130118, China; 3 Center of Excellence in Fungal Research, Mae Fah Luang University, Chiang Rai 57100, Thailand; avnishbhunjun@gmail.com (C.S.B.); kdhyde3@gmail.com (K.D.H.); 4 School of Science, Mae Fah Luang University, Chiang Rai 57100, Thailand; 5 The Center for Yunnan Plateau Biological Resources Protection and Utilization, College of Biological Resource and Food Engineering, Qujing Normal University, Qujing 655011, China; saowaluckfai@gmail.com; 6 China Innovative Institute for Plant Health, Zhongkai University of Agriculture and Engineering, Guangzhou 510225, China; 7 Jiaxing Key Laboratory for New Germplasm Breeding of Economic Mycology, Jiaxing 314000, China

**Keywords:** new taxa, new record, *Pleosporales*, saprobic fungi, taxonomy, *Xanthoceras sorbifolium*

## Abstract

*Leptosphaeriaceae*, a diverse family in the order *Pleosporales*, is remarkable for its scleroplectenchymatous or plectenchymatous peridium cells. Four *Leptosphaeriaceae* species were discovered and studied during the investigation of saprobic fungi from plant substrates in China. Novel taxa were defined using multiloci phylogenetic analyses and are supported by morphology. Based on maximum likelihood (ML) and Bayesian inference (BI) analyses, these isolates represent three novel taxa and one new record within *Leptosphaeriaceae*. A new genus, *Angularia*, is introduced to accommodate *Angularia xanthoceratis*, with a synopsis chart for 15 genera in *Leptosphaeriaceae.* This study also revealed a new species, *Plenodomus changchunensis*, and a new record of *Alternariaster centaureae-diffusae*. These species add to the increasing number of fungi known from China.

## 1. Introduction

**Figure 1:**
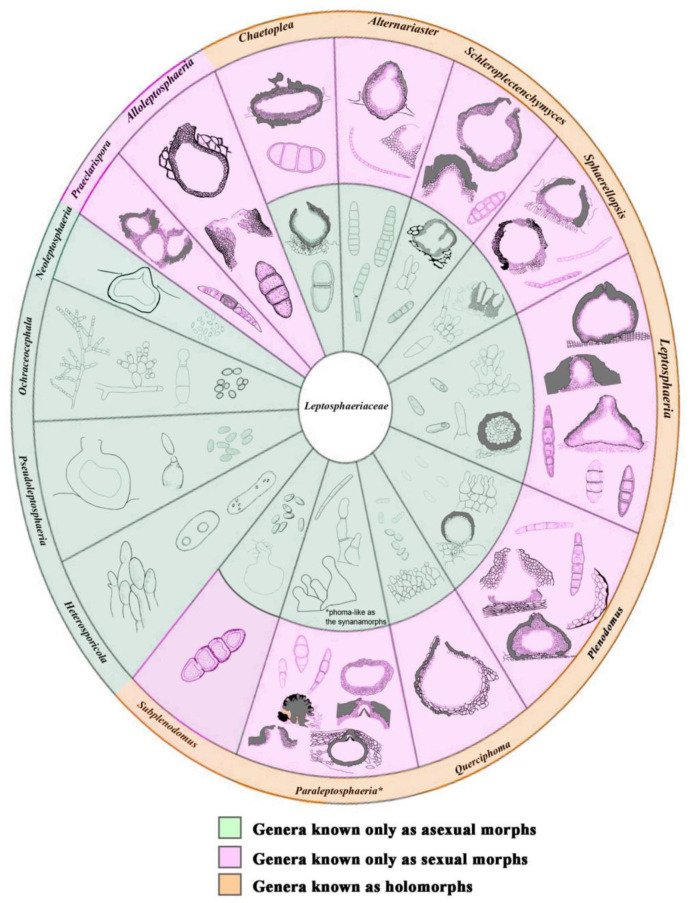
Morphology of ascomata, conidiomata, ascospores, and conidiogenous cells; and conidia of 15 genera in *Leptosphaeriaceae*. Asterisk (*) indicates the genera with synanamorphs asexual characters.

*Leptosphaeriaceae* is an important group of fungi in the order *Pleosporales* [1,2,3,4,5,6]. *Leptosphaeriaceae* was segregated from *Pleosporaceae* by Barr (1987) and was typified by *Leptosphaeria* Ces. & De Not. [1,2,3]. This family is characterized by conical or globose ascomata, scleroplectenchymatous or plectenchymatous peridium cells, cylindrical to oblong pedicellate asci, and septate reddish-brown or yellowish-brown ascospores (Figure 1) [2,4,7,8,9,10,11,12,13,14]. Although *Leptosphaeriaceae* is similar to *Phaeosphaeriaceae*, the peridium structure is morphologically distinguishable [15]. Most *Leptosphaeriaceae* species occur abundantly on dicotyledons, and the asexual morph can be coelomycetous (coniothyrium-like or phoma-like) or hyphomycetous [12,16,17]. Members of *Leptosphaeriaceae* are saprobes, hemibiotrophs, and pathogens [18,19,20,21,22]. Five genera *Curreya*, *Didymolepta*, *Heptamaeria*, *Leptosphaeria*, and *Ophiobolus* were previously included in the family [1]. Hyde et al. [2] accepted *Heterosporicola*, *Leptosphaeria*, *Neophaeosphaeria*, *Paraleptosphaeria*, *Plenodomus*, and *Subplenodomus* in the family by integrating molecular data. Simmons [23] introduced *Alternariaster* to accommodate *Alternariaster helianthi* (=*Alternaria helianthi*) as the first hyphomycetous record for *Leptosphaeriaceae*. Trakunyingcharoen et al. [24] subsequently introduced *Sphaerellopsis* from *Dianthus caryophyllus* and *Vachellia karroo*. The family was revised based on morphological characteristics and phylogenetic evidence, and ten genera were accepted [4]. Several other genera have also been added to *Leptosphaeriaceae*, such as *Heterosporicola*, *Ochraceocephala*, *Querciphoma*, *Sclerenchymomyces*, and *Praeclarispora* [8,12,13,14].

Preuss (1851) introduced *Plenodomus*, which was typified by *P. rabenhorstii* [25]. The *Plenodomus* species belong to *Leptosphaeriaceae* and are one of the members with phoma-like taxa [2,5,17]. The type material of *P. rabenhorstii* was lost, and therefore *P. lingam* (Tode) Hohn. (Sexual morph: *Leptosphaeria maculans* (Desm.) Ces. & De Not.) was replaced as the type species of *Plenodomus* [26]. Phoma-like taxa were previously classified into nine sections including *Plenodomus* based on morphological characteristics [27,28]. de Gruyter et al. [29] determined that the *Plenodomus* section was distinct from *Phoma sensu stricto* based on phylogenetic analyses and classified *Phoma* under *Didymellaceae*. The *Plenodomus* species are the causal agents of diverse diseases on different plants throughout the world [30,31]. *Plenodomus* species are also isolated as saprobes on dead branches and stems of plants [17].

*Alternariaster* was introduced by Simmons [23] to accommodate *Alternaria helianthi*, a causal agent of leaf spots of *Helianthus annuus* (sunflower) worldwide [23,32,33]. This genus was segregated from *Alternaria* based on different conidial morphology. Alves et al. [8] confirmed that *Alternariaster* is a member of *Leptosp haeriraceae* and is distinct from *Alternaria* (*Pleosporaceae*). Four species have been reported in *Alternariaster*, including *A. bidentis* [16], *A. centaureae-diffusae* [4], *A. helianthi* [23], and *A. trigonosporus* [2]. *Alternariaster helianthi* has been reported worldwide as a pathogen of leaf spots on sunflowers, and *Alternariaster bidentis* was reported only from Brazil, whereas *Alternariaster centaureae-diffusae* and *Alternariaster trigonosporus* were reported from Russia [2,4]. This genus has been associated with *Bidens sulphurea*, *Centaurea diffusa*, *Cirsium* sp., and *Helianthus annuus* [2,4,16,23].

In this study, we introduce one new genus (*Angularia*), two new species (*Angularia xanthoceratis* and *Plenodomus changchunensis*), and one new record of *Alternariaster centaureae-diffusae* collected from China. The species were compared morphologically with other *Leptosphaeriaceae* species. Phylogenetic analyses were performed to confirm the taxonomic position based on maximum likelihood and Bayesian inference of combined LSU, SSU, ITS, and *tub*2 datasets.

## 2. Materials and Methods

### 2.1. Sample Collection and Isolation

The dried stems of *Xanthoceras sorbifolium* Bunge, *Poaceae*, and *Clematis* L. were collected from Changchun, Jilin Province and Kunming, Yunnan Province, China. The samples were preserved in plastic bags with labels describing location, date, host, and collection details. Pure fungal colonies were obtained using single spore isolation [34]. Germinating spores were transferred aseptically to potato dextrose agar (PDA), and the cultures were incubated at 25 °C. The specimens and pure cultures were deposited in the Herbarium of Mycology, Jilin Agricultural University (HMJAU), Changchun, China and International Cooperation Research Center of China for New Germplasm Breeding of Edible Mushrooms Culture Collection (CCMJ), respectively. The new taxa were registered in Mycobank [35].

### 2.2. Morphological Observation

Ascomata and conidiomata characteristics of the hosts were observed using a Zeiss Stemi 2000C stereomicroscope equipped with a Leica DFC450C digital camera (Leica, Wetzlar, Germany). Hand sections of the ascomata were carried out, and the sections were mounted on a slide with a drop of distilled water. Morphological characteristics were observed and photographed using a Zeiss AX10 light microscope equipped with an Axiocam 506 digital camera. Microscopic measurements were carried out using the ZEN 3.4 (blue edition) program (ZEISS, Jena, Germany). Adobe Photoshop CC2020 (Adobe Systems, San Jose, CA, USA) was used to process the images.

### 2.3. DNA Extraction, PCR Amplification and Sequencing

DNA was extracted from pure culture using a NuClean PlantGen DNA Kit (CWBIO, China) following the manufacturer’s instructions. Polymerase chain reaction (PCR) was used for the amplification of the large subunit (LSU), small subunit (SSU), internal transcribed spacer regions (ITS), β-tubulin (*tub*2), and the RNA polymerase II second largest subunit (*rpb*2). The LSU gene was amplified with the primers LROR and LR5 [36]; the SSU gene was amplified with the primers NS1 and NS4 [37]; the nuclear ITS was amplified with the primers ITS5 and ITS4 [37]; the *tub*2 gene was amplified with primers T1 and Bt2b [38]; and the *rpb*2 gene was amplified with primers RPB2-5f2 and fRPB2-7cr [39]. The amplification reactions were performed using 20 μL PCR mixtures containing 9 μL sterilized water, 10 μL of 2 × Es Taq MasterMix (Dye), 0.3 μL (10 μM) of forward and reverse primers, and 0.4 μL (200 ng/μL) of DNA template. The PCR conditions for LSU, SSU, ITS, and *tub*2 were as follows: 94 °C for 5 min, then 35 cycles of denaturation at 94 °C for 30 s, annealing at 53 °C for 45 s, elongation at 72 °C for 90 s, and a final extension at 72 °C for 10 min. All the PCR products were visualized on 1% agarose gels stained with standard DNA dye.

### 2.4. Phylogenetic Analysis

The sequence data were assembled using BioEdit v.7.2.5 [40] The closest matches for the new strains were obtained by using BLASTn searches (accessed on 13 December 2021, http://www.blast.ncbi.nlm.nih.gov/), and reference sequence data were downloaded from recent publications (Table 1) [41,42]. *Didymella exigua* (CBS 183.55) and *D. rumicicola* (CBS 683.79) were selected as the outgroup taxa. The sequences were aligned by using MAFTT version 7 (accessed on 7 March 2022, mafft.cbrc.jp/alignment/server) [43], and ambiguous nucleotides were manually adjusted by visual examination in AliView where necessary [44]. Leading or trailing gaps beyond the primer binding site were trimmed from the alignments prior to phylogenetic analyses, and the alignment gaps were treated as missing data.

Phylogenetic analyses of individual and multiloci phylogenetic analyses (ITS, LSU, SSU, and *tub*2) were performed to determine the phylogenetic placement of the isolated taxa. Maximum likelihood analysis was performed using RAxML-HPC2 on XSEDE on the CIPRES web portal (accessed on 7 March 2022, http://www.phylo.org/portal2/) [45,46,47]. The GTR + GAMMA model of nucleotide evolution was used for the datasets, and RAxML rapid bootstrapping of 1000 replicates was performed. The best-fit evolutionary models for individual and combined datasets were estimated under the Akaike Information Criterion (AIC) using jModeltest 2.1.10 on the CIPRES web portal for posterior probability [48]. The GTR model was the best model for all the datasets. Bayesian inference analyses were performed using MrBayes v. 3.2.6 on the CIPRES web portal [49]. Simultaneous Markov chains were run for seven million generations, and trees were sampled every 100th generations.

FigTree v. 1.4 [50] was used to visualize phylogenetic trees. The phylogram was edited by using Adobe Illustrator CS v. 6. All newly generated sequences were deposited in GenBank. All the alignments and trees were deposited in TreeBASE (Submission ID: 29394 and 29395).

## 3. Results

### 3.1. Phylogenetic Analyses

**Figure 2:**
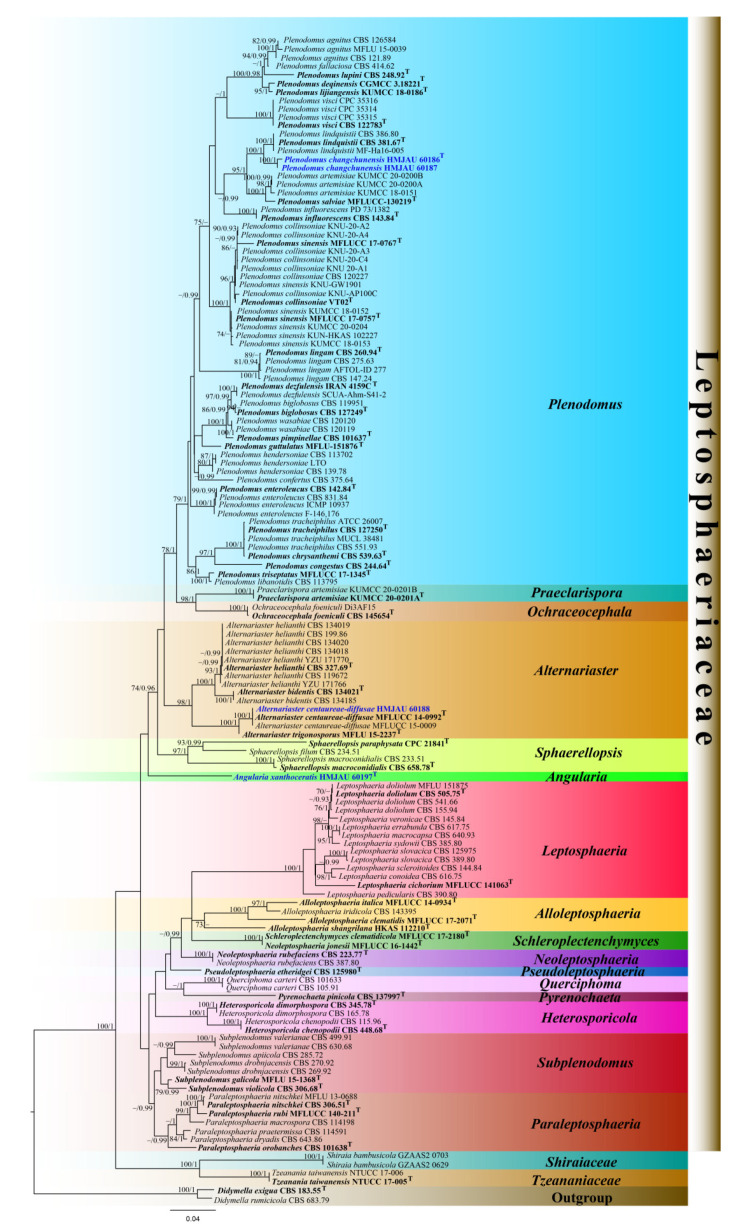
The best scoring RAxML tree of *Leptosphaeriaceae* based on a concatenated ITS, LSU, SSU, and *tub*2 datasets. The tree is rooted with *Didymella exigua* (CBS 183.55) and *D. rumicicola* (CBS 683.79). RAxML bootstrap support values ≥70% (ML, left) and Bayesian posterior probabilities ≥0.90 (BPP, right) are shown near the nodes. The new isolates are in blue. The type strains are in bold and marked with ^T^.

The combined LSU, SSU, ITS, and *tub*2 datasets comprised 138 strains, including our newly sequenced strains. Multiloci data were concatenated, which comprised 2958 characteristics, including gaps (ITS: 1–643, LSU: 644–1509, SSU: 1510–2573, and *tub*2: 2574–2970). The RAxML analysis yielded a best scoring tree (Figure 2) with a final ML optimization likelihood value of −19828.46. The matrix had 928 distinct alignment patterns, with 39.78% undetermined characteristics or gaps. Estimated base frequencies were as follows: A = 0.240304, C = 0.229231, G = 0.271334, and T = 0.259131; substitution rates AC = 1.321448, AG = 2.815733, AT = 1.680962, CG = 0.694608, CT = 5.562821, and GT = 1.000000; proportion of invariable sites I = 0.704486; and gamma distribution shape parameter α = 0.555544. Phylogenetic trees generated from the Bayesian and maximum likelihood analyses had similar topologies (Figure 2 and Figure S1). However, in the Bayesian analysis, *Alloleptosphaeria shangrilana* did not cluster within the *Alloleptosphaeria* clade, but was sister to the *Schleroplectenchymyces* species with low support (0.72 BPP). The MLBP values (left) and BPP values (right) are provided near each node (Figure 2). For the Bayesian analysis, a total of 10,338 trees were sampled after the 20% burn-in with a stop value of 0.009971.

*Leptosphaeriaceae* was strongly supported in the maximum likelihood and Bayesian analyses (100% ML/1.00 BPP). Within *Leptosphaeriaceae*, *Heterosporicola*, *Leptosphaeria*, *Neoleptosphaeria*, *Ochraceocephala*, *Praeclarispora*, *Querciphoma*, and *Schleroplectenchymyces* strongly supported clades (100% ML/1.00 BPP) were formed. *Alternariaster* (98% ML/1.00 BPP) and *Sphaerellopsis* (97% ML/1.00 BPP) formed strongly supported clades, while *Alloleptosphaeria* and *Plenodomus* were only moderately supported in the maximum likelihood analyses (73% ML and 79% ML, respectively). The newly introduced genus formed an independent lineage basal to *Sphaerellopsis* with 35% ML/0.81 BPP support. A new genus *Angularia* is therefore introduced within *Leptosphaeriaceae*. The newly generated taxa *Plenodomus changchunensis* (HMJAU 60186 and HMJAU 60187) clustered with *Plenodomus lindquistii* with 100% ML/1.00 BPP support, while the strain HMJAU 60188 formed a strongly supported clade with *Alternariaster centaureae-diffusae* taxa (Figure 2).

### 3.2. Taxonomy

***Angularia*** R. Xu, Phukhams. & Y. Li, *gen. nov.*

**MycoBank Number:** 843307.

**Etymology:** referring to the angular peridium of the type species.

**Description:***Saprobic* on decaying wood or herbaceous plant material in terrestrial habitats. **Sexual morph**: Undetermined. **Asexual morph**: *Conidiomata* pycnidial, solitary, sometimes aggregated, uniloculate, immersed in host substrate, dark brown to brown, globose, coriaceous. *Ostioles* absent. *Conidiomatal wall* thick-walled, multilayered, scleroplectenchymatous cells thick at base, composed of *textura angularis*, lined with a thick hyaline layer bearing conidiogenous cells. *Conidiophores* reduced to conidiogenous cells. *Conidiogenous cells* enteroblastic, phialidic, determinate, discrete, subcylindrical to truncate, smooth-walled, hyaline, arising from the inner layers of conidiomata. *Conidia* fusiform, truncate at both ends, aseptate, hyaline, smooth.

**Type species: *Angulariaxanthoceratis*** R. Xu, Phukhams. & Y. Li.

**Figure 3:**
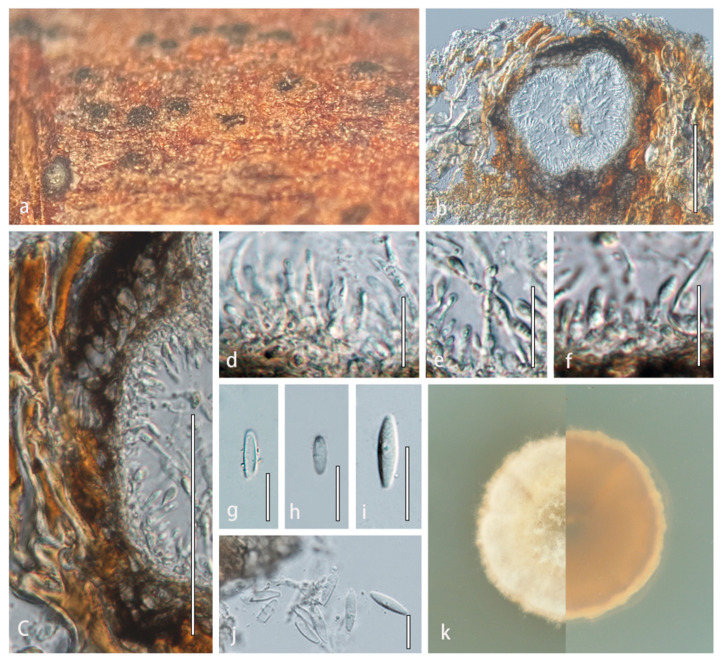
*Angularia xanthoceratis* (HMJAU 60197, **holotype**). (**a**) Appearance of conidiomata on host substrate. (**b**) Vertical section of conidioma. (**c**) Section of conidioma wall. (**d**–**f**) Conidiogenous cells and conidia. (**g**–**j**) Conidia. (**k**) Culture characteristics on PDA after two weeks at 25 °C. Scale bars: (**b**) = 100 µm; (**c**) = 50 µm; and (**d**–**j**) = 20 µm.

**Notes**: *Angularia* is introduced for a strongly supported lineage comprising *Angularia xanthoceratis* (1.00 BPP, Figure 2). *Angularia* formed a distinct lineage to *Alternariaster*, *Ochraceocephala*, *Plenodomus*, *Praeclarispora* and *Sphaerellopsis* based on multiloci phylogenetic analyses. For individual loci, *Angularia* formed a sister clade distinct from *Heterosporicola* (ITS) and formed a sister clade distinct from *Pseudoleptosphaeria_etheridgei* (LSU). *Leptosphaeriaceae* species are remarkable for having superficial to semi-immersed, shiny ascomata or conidiomata, with thick, multilayers of scleroplectenchymatous or pseudoparenchymatous tissue types [4]. The fungus has semi-immersed to immersed conidiomata, black, with a multilayer scleroplectenchymatous-type tissue (Figure 3). *Angularia* is similar to *Plenodomus* and *Alternariaster* in having peridium with scleroplectenchymatous cells [4]. *Angularia* is also similar to *Plenodomus* and *Sphaerellopsis* in having *textura angularis* cells in the conidiomatal wall [4,24]. However, *Angularia* and *Ochraceocephala* differ substantially in morphology. *Ochraceocephala* has long and branched conidiophores, and the branching is commonly irregularly verticillate, while the conidiophores of *Angularia* are reduced to conidiogenous cells. *Ochraceocephala* has hyaline to yellowish, mostly sand to olive yellow, and mostly globose to subglobose conidia, while *Angularia* has hyaline and fusiform conidia; the conidia are smaller than in our new genus (4.8 vs. 18.7 × 3.6 vs. 5.4 μm).

***Angulariaxanthoceratis*** R. Xu, Phukhams. & Y. Li, sp. *nov.* (Figure 3).

**MycoBank Number**: 843308.

**Etymology**: referring to the host genus, *Xanthoceras.*

**Holotype**: HMJAU 60197.

**Description**: Saprobic on dead stems of *Xanthoceras sorbifolium*. **Sexual morph**: Undetermined. **Asexual morph**: *Conidiomata* 180–220 × 195–224 μm ($\bar{x}$ = 200 × 210 μm, *n* = 5), pycnidial, solitary, aggregated, uniloculate, immersed in host substrate, globose, thick-walled, subcoriaceous to coriaceous at the outer layers, dark brown to brown, without distinct ostioles. *Ostioles* absent. *Conidiomatal wall* 20–46 μm wide, thick, multilayered, scleroplectenchymatous cells, outer layer composed of 6–8 layers of dark brown to brown cells of *textura angularis*, lined with a thick hyaline layer bearing conidiogenous cells. *Conidiophores* reduced to conidiogenous cells. *Conidiogenous cells* 7.8–20.8 × 1.7–3.5 μm ($\bar{x}$ = 14.3 × 2.6 μm, *n* = 20), enteroblastic, phialidic, determinate, discrete, subcylindrical to truncate, smooth-walled, hyaline, arising from the inner layers of conidiomata. *Conidia* 13–24.5 × 4–7 μm ($\bar{x}$ = 18.7 × 5.4 μm, *n* = 30), fusiform, truncate at both ends, aseptate, hyaline, smooth-walled.

**Culture characteristics**: Colonies on PDA reaching 20 mm in diameter after 2 weeks at 25 °C. Cultures from above, dome-shaped in the center, milky white radiating outward, dense, round, creeping hyphae; reverse dark at the center, light orange radiating outward.

**Material examined**: CHINA, Jilin Province, Changchun, on dead stem of *Xanthoceras sorbifolium* (*Sapindaceae*), 15 September 2021, Rong Xu, HMJAU 60197 (**holotype**); extype living culture, CCMJ5013.

**GenBank accession numbers**: LSU = OM295682, SSU = OM295681, ITS = OM295683, and *tub*2 = OM304358

**Notes**: *Angularia xanthoceratis* is distinct from the closely related *Sphaerellopsis* species in conidial characteristics (Figure 3). *Angularia xanthoceratis* has fusiform, smooth-walled, hyaline, aseptate conidia, which are truncate at both ends, while *Sphaerellopsis* has fusoid-ellipsoidal, occasionally Y-shaped or digitate, subcylindrical to ellipsoid or globose, pale brown, 0–1(−3)-euseptate conidia [24]. In a BLASTn search, the LSU sequence of *Angularia xanthoceratis* was 99.55% similar to *Leptosphaeria etheridgei* (CBS 125980) with 96% query cover which translates to 95.6% similarity. The ITS region was 97.44% similar to *Leptosphaeria* sp. (Ct-BC63) with 82% query cover which translates to 79.9% similarity. A pairwise comparison of the ITS region revealed 119 bases pair differences (18.39%) between *A*. *xanthoceratis* and *Sphaerellopsis macroconidialis*, while the *tub*2 region was 98 bases pair different (24.62%).

**Figure 4:**
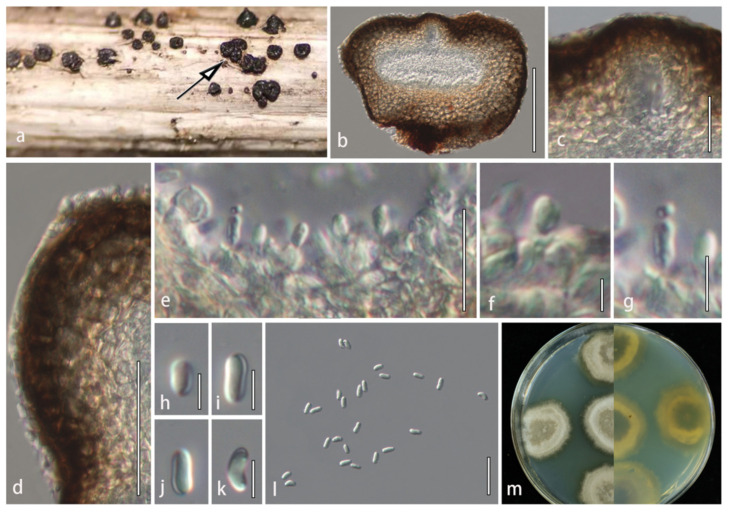
*Plenodomus changchunensis* (HMJAU 60186, holotype). (**a**) Appearance of conidiomata on host substrate; black arrow indicates the conidiomata of *P. changchunensis* on the host. (**b**) Vertical section of conidioma. (**c**) Ostiolar canal. (**d**) Section of conidioma wall. (**e**–**g**) Conidiogenous cells and conidia. (**h**–**l**) Conidia. (**m**) Culture characteristics on PDA after three weeks at 25 °C. Scale bars: (**b**) = 100 µm; (**c**,**e**,**l**) = 20 µm; (**d**) = 50 µm; and (**f**–**k**) = 5 µm.

***Plenodomus changchunensis*** R. Xu, Phukhams. & Y. Li, *sp. nov*. (Figure 4)

**MycoBank Number:** 843304

**Holotype**: HMJAU 60186

**Etymology**: referring to Changchun city where this fungus was collected.

**Description**: Saprobic on dead stems of *Poaceae*. **Sexual morph**: Undetermined. **Asexual morph**: *Conidiomata* 163–192 × 193–245 μm (*x* = 175 × 207 μm, *n* = 5), pycnidial, solitary or in groups of 2–5, erumpent, aggregated, globose to subglobose, depression in the middle, thick-walled, subcoriaceous to coriaceous at the outer layers, dark brown to black, ostiolate. *Ostioles* 20–45 μm, central, papillate, ovoid, filled with short periphyses. *Conidiomatal wall* 24–48 μm wide, thick, multilayered, outer layer composed of 8–10 layers of dark brown to brown cells of *textura angularis*, lined with a thick hyaline layer bearing conidiogenous cells. *Conidiophores* reduced to conidiogenous cells. *Conidiogenous cells* 2.8–5.8 × 1.5–2.8 μm (*x* = 4.1 × 2 μm, *n* = 30), enteroblastic, phialidic, determinate, smooth-walled, hyaline. *Conidia* 5–7.6 × 2–3.4 μm (*x* = 6.2 × 2.7 μm, *n* = 50), oblong or oval, slightly curved toward the ends, rounded ends, aseptate, hyaline, smooth-walled.

**Culture characteristics**: Colonies on PDA reaching 30 mm diam. after 3 weeks at 25 °C. Cultures from above, gray in the center, milky white radiating outward, dense, circular, creeping hyphae, grayish-green at the margins; reverse dark at the center, milky white radiating outward. Yellow pigmentation diffused into the media.

**Material examined**: CHINA. Jilin Province: Changchun, on dead twigs of *Poaceae* sp., 20 May 2021, C. Phukhamsakda, HMJAU 60186 (holotype); extype living culture, CCMJ5011; HMJAU 60187 (isotype), ex-isotype living culture, CCMJ5012.

**GenBank accession numbers**: LSU = OL897174, SSU = OL984031, ITS = OL996123, and *tub*2 = OM009247

**Notes**: *Plenodomus changchunensis* (CCMJ5011 and CCMJ5012) formed a sister clade distinct from *Plenodomus lindquistii* with 99% ML/1.00 BPP support based on phylogenetic analysis of the concatenated ITS, LSU, SSU, and *tub*2 datasets (Figure 2). *Plenodomus changchunensis* is similar to *P. lindquistii* in the size of conidia [51]. This species can be distinguished from *P. lindquistii* (CBS 381.67) by 34 nucleotides in the ITS region (34/643 in the ITS region and 0/866 in the LSU region). In the BLASTn search, the closest match to the LSU and ITS sequences of *P. changchunensis* were 100% and 89.57% similar to *Leptosphaeria* sp. (PHY-30) and *P. lindquistii* (MCN535002) with 95% query cover which translates to a 95% and 85.1% similarity, respectively. *Plenodomus changchunensis* was found associated with a grass near the water resources in temperate regions. Therefore, this fungus is introduced as a novel species.

**Figure 5:**
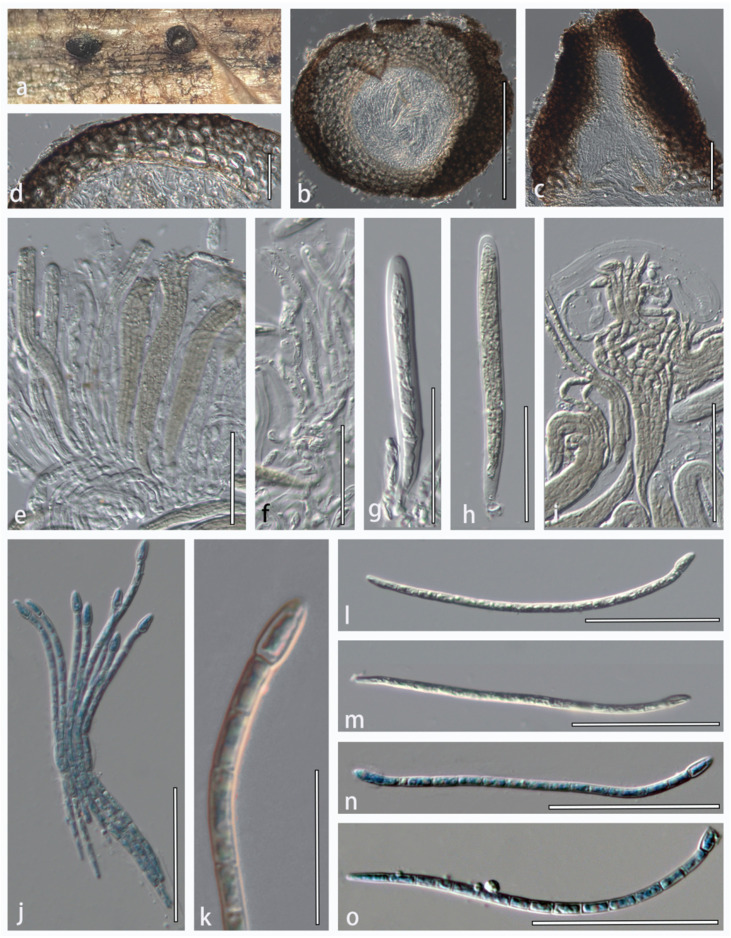
*Alternariaster centaureae-diffusae* (HMJAU 60188). (**a**) Appearance of ascomata on host substrate. (**b**) Vertical section of ascoma. (**c**) Ostiole with periphyses. (**d**) Close-up of peridium. (**e**,**g**,**h**) Immature and mature asci. (**f**) Pseudoparaphyses. (**i**,**j**) Fissitunicate asci. (**k**) Top part of ascospore. (**l**–**o**) Ascospores. (**j**,**n**,**o**) Ascospores were stained in cotton blue. Scale bars: (**b**) = 200 μm; (**c**,**d**,**f**–**j**,**l**–**o**) = 50 μm; (**e**) = 100 μm; and (**k**) = 20 μm.

***Alternariaster centaureae-diffusae*** R.H. Perera, Bulgakov, Ariyawansa & K.D. Hyde, in Fungal Diversity, 74: 32 (2015), new host record and new geological record (Figure 5)

**Index Fungorum Identifier:** IF551462

**Description:** Saprobic on dried stems of *Clematis* sp. **Sexual morph**: *Ascomata* 170–360 × 146–290 μm diam., solitary or in groups of 2–10, erumpent, semi-immersed or nearly superficial, uniloculate, globose to subglobose, coriaceous, black, ostiolate. *Ostiole* papillate, black, filled with periphyses. *Periphyses* aseptate, with a blunt apex, hyaline. *Peridium* 40–75 μm wide (*x* = 57.5 μm, *n* = 10), comprising thick-walled cells of *textura globularis*, inner layer composed of flattened cells of *textura angularis*, 5–10 rows of scleroplectenchymatous cells, outer layer thick, black. *Hamathecium* 2.5–3.8 μm wide, dense, distinctly septate, branched, cellular pseudoparaphyses, hyaline, embedded in a gelatinous matrix. *Asci* 110–140 × 10–14 μm (*x* = 125 × 12 μm, *n* = 20), 8-spored, bitunicate, fissitunicate, cylindrical to cylindric-subclavate, with a short bulbous pedicel, rounded at the apex. *Ascospores* 80–138 × 2.3–4.3 μm (*x* = 109 × 3.3 μm, *n* = 40), fasciculate, filiform, 14–16-septate, constricted at the apical septum, apical cell swollen, conical, yellowish-brown, smooth-walled, with a mucilaginous cap. **Asexual morph:** Undetermined.

**Material examined**: CHINA, Yunnan Province, dead aerial branch of *Clematis* spp., 24 April 2021, (HMJAU 60188).

**Host associations**: *Centaurea diffusa*, *Clematis* spp. ([4] and this study).

**GenBank accession numbers**: LSU = OL897175, SSU = OL891810, ITS = OL996125, and *tub*2 = OL898721

**Notes**: *Alternariaster centaureae-diffusae* was originally described from the dead stems of *Centaurea diffusa* Lam. in Russia [4]. The new isolate (HMJAU 60188) has similar morphology to the type strain of *A. centaureae-diffusae* (MFLU 15–1521) in having fasciculate, filiform, constricted at the apical septum, conical, yellowish-brown ascospores with swollen apical cell [4]. A pairwise comparison of the sequences of the new isolate (HMJAU 60188) with the type species of *A. centaureae-diffusae* revealed minor differences. The new isolate clustered in the same clade as the type strain of *A. centaureae-diffusae* (Figure 2). Therefore, we report *A. centaureae-diffusae* on *Clematis* spp. as a new host and new geological record.

## 4. Discussion

Molecular biology has helped to elucidate the phylogenetic relationships among members of *Dothideomycetes*, particularly among several phoma-like taxa [13,52]. Multi-loci analyses based on LSU, SSU, ITS, *tub*2, *rpb*2, and *tef*-1 sequences have been widely used to define species boundaries in *Leptosphaeriaceae* and other families of *Dothideomycetes* [13,52,53]. We carried out phylogenetic analyses with a concatenated dataset of five loci (ITS, LSU, SSU, *tub*2, and *rbp*2) for *Leptosphaeriaceae* members. The final alignment included 138 strains representing 132 ingroup taxa and six outgroup strains. However, the *Plenodomus* species were polyphyletic and mixed with *Alternariaster*, *Ochraceocephala*, and *Praeclarispora* taxa. It is often encouraged to use additional taxon-specific secondary barcode loci to delineate taxa. We therefore compared the phylogenetic informativeness of *tub*2 (52 sequences translated to 37.7%) and *rpb*2 (46 sequences translated to 33.3%) sequences of *Leptosphaeriaceae*. Our study shows that the polyphyletic topology of the *Plenodumus* group is due to the *rpb*2 gene (Figures S2–S4). This could be due to a lack of *rpb*2 barcodes in several related taxa, but the *rpb*2 gene can be useful for delineation at the genus level [12,41]. In contrast, using the *tub*2 gene provides a better resolution at the species level within the genera (Figure 2). Therefore, we performed phylogenetic analyses of *Leptosphaeriaceae* species with a concatenated dataset of ITS, LSU, SSU, and *tub*2 loci. Three new species of *Leptosphaeriaceae* were revealed from China based on multilocus phylogeny combined with morphology.

The phylogeny from our analyses is similar to several previous studies [4,12,13]. The *Leptosphaeriaceae* taxa clustered in fifteen clades based on the ITS, LSU, SSU, and *tub*2 datasets. A novel genus *Angularia* is also introduced in *Leptosphaeriaceae* to accommodate a new species, *A*. *xanthoceratis*. Conidial characteristics are the primary morphological characteristics that distinguish *Angularia* from the allied genus *Sphaerellopsis* (Figure 1). *Plenodomus* formed a separate clade, sister to *Ochraceocephala*, and revealed a novel species *P. changchunensis* with strong support. Many new genera have been introduced in *Leptosphaeriaceae* [2,4,8,12,13,14,23], which indicates that this family has a high degree of fungal diversity and distribution.

*Plenodomus lingam* was chosen to be the representative type species of *Plenodomus* over *P. rabenhorstii* Preuss [14,54]. There are 36 epithets listed under *Plenodomus* in Species Fungorum (2022) and 107 epithets in MycoBank. The host specificity of *Plenodomus* has not yet been clarified as species have been recorded from various plant families (*Asteraceae*, *Fabaceae*, *Lamiaceae*, and *Liliaceae*) [9]. In our study, *P. changchunensis* was found on *Poaceae*, which suggests that the *Leptosphaeriaceae* species are widely associated with many types of substrates. Members of *Plenodomus* appear to be cosmopolitan, as they have been recorded in both temperate and tropical countries (China, Greece, France, Japan, Netherlands, Peru, and Spain) [55].

*Alternariaster centaureae-diffusae* has been isolated from *Centaurea diffusa* Lam. (*Asteraceae*) in Shakhty city, Rostov region, Russia [4]. In this study, it was isolated from *Clematis* spp. (*Ranunculaceae*) in Kunming, Yunnan province, China. Therefore, our study extended the host range of *A. centaureae-diffusae* even though the environment of the two cities is different (temperate and subtropical). Therefore, we speculate that this species could be found in different environments and hosts [56].

Fungal diversity and taxonomy are constantly changing, necessitating a continuous assessment [57,58,59]. It is especially significant where taxa are described from genera that usually accommodate pathogens [60,61]. For example, *Plenodomus* and *Alternariaster* are the causal agents of blackleg disease and leaf spots of *Helianthus annuus* (sunflower) worldwide [31,32,62,63]. The discovery of novel species in a pathogenic genus could also indicate the discovery of emerging pathogens that can cause damage to economically important crops [64,65]. The formation of new fungi species has been reported to be intricately linked to their evolutionary relationships and ecological roles [20]. These phenomena can also occur when species are associated with different hosts and environments, as in the case of *A. centaureae-diffusae* in this study. The presence of the *Alternariaster* and *Plenodomus* species in different substrates reflects their ecological importance. Further studies focusing on fungal diversity from different niches are needed to understand the relationships between these organisms in ecosystems.

## Figures and Tables

**Table 1:** Taxa and GenBank accession numbers used in the phylogenetic analyses. The extypes are shown in bold, and newly generated sequences are shown in blue.

Species	Host	Strain/Isolate	GenBank Accession Numbers			
			ITS	LSU	SSU	*tub*2
***Alloleptosphaeria clematidis***	***Clematis subumbellata***	**MFLUCC 17-2071**	**MT310604**	**MT214557**	**MT226674**	**_**
***All. iridicola***	***Iris* sp.**	**CBS 143395**	**MH107919**	**MH107965**	**_**	**_**
***All. italica***	**_**	**MFLUCC 14-0934**	**KT454722**	**KT454714**	**_**	**_**
***All. shangrilana***	**_**	**HKAS: 112210**	**MW431059**	**MW431315**	**MW431058**	**_**
***Alternariaster bidentis***	***Bidens sulphurea***	**CBS 134021**	**KC609333**	**KC609341**	**_**	**_**
*Alt. bidentis*	*Bidens sulphurea*	CBS 134185	KC609334	KC609342	_	_
***Alt. centaureae-diffusae***	***Centaurea diffusa Lam.***	**MFLUCC 14-0992**	**KT454723**	**KT454715**	**KT454730**	**_**
*Alt. centaureae-diffusae*	*Centaurea diffusa*	MFLUCC 150009	KT454724	KT454716	KT454731	_
***Alt. centaureae-diffusae***	***Clematis* spp.**	**HMJAU 60188**	**OL996125**	**OL897175**	**OL891810**	**OL898721**
*Alt. helianthi*	_	YZU 171766	MZ702726	_	_	_
*Alt. helianthi*	_	YZU 171770	MZ702727	_	_	_
***Alt. helianthi***	***Helianthus annuus***	**CBS 327.69**	**KC609335**	**KC584369**	**KC584627**	**_**
*Alt. helianthi*	*Helianthus annuus*	CBS 199.86	KC609336	KC609343	_	_
*Alt. helianthi*	*Helianthus* sp.	CBS 119672	KC609337	KC584368	KC584626	_
*Alt. helianthi*	*Helianthus annuus*	CBS 134018	KC609338	KC609344	_	_
*Alt. helianthi*	*Helianthus annuus*	CBS 134019	KC609339	KC609345	_	_
*Alt. helianthi*	*Helianthus annuus*	CBS 134020	KC609340	KC609346	_	_
***Alt. trigonosporus***	***Cirsium* sp.**	**MFLU 15-2237**	**KY674857**	**KY674858**	**_**	**_**
***Angularia xanthoceratis***	***Xanthoceras sorbifolium***	**HMJAU 60197**	**OM295683**	**OM295682**	**OM295681**	**OM304358**
***Didymella exigua***	***Rumex arifolius***	**CBS 183.55**	**GU237794**	**EU754155**	**EU754056**	**GU237525**
***D. rumicicola***	***Rumex obtusifolius***	**CBS 683.79**	**KT389503**	**KT389721**	**_**	**KT389800**
***Heterosporicola chenopodii***	***Chenopodium album***	**CBS 448.68**	**FJ427023**	**EU754187**	**EU754088**	**_**
*H. chenopodii*	*Chenopodium album*	CBS 115.96	JF740227	EU754188	EU754089	_
*H. dimorphospora*	*Chenopodium quinoa*	CBS 165.78	JF740204	JF740281	JF740098	_
***H. dimorphospora***	***Chenopodium quinoa***	**CBS 345.78**	**JF740203**	**GU238069**	**GU238213**	**_**
***Leptosphaeria cichorium***	***Cichorium intybus***	**MFLUCC 14-1063**	**KT454720**	**KT454712**	**KT454728**	**_**
*L. conoidea*	*Lunaria annua*	CBS 616.75	JF740201	JF740279	_	KT389804
*L. doliolum*	*Phlox paniculata*	CBS 155.94	JF740207	JF740282	_	JF740146
*L. doliolum*	_	MFLU: 151875	KT454727	KT454719	KT454734	_
*L. doliolum*	*Rudbeckia* sp.	CBS 541.66	JF740206	JF740284	_	JF740145
***L. doliolum***	***Urtica dioica***	**CBS 505.75**	**JF740205**	**GQ387576**	**GQ387515**	**JF740144**
*L. errabunda*	*Solidago* sp.	CBS 617.75	JF740216	JF740289	_	JF740150
*L. macrocapsa*	*Mercurialis perennis*	CBS 640.93	JF740237	JF740304	_	JF740156
*L. pedicularis*	*Pedicularis* sp.	CBS 390.80	JF740224	JF740294	_	JF740155
*L. scleroitoides*	*Medicago sativa*	CBS 144.84	JF740192	JF740269	_	_
*L. slovacica*	*Ballota nigra*	CBS 125975	JF740248	JF740316	_	_
*L. slovacica*	*Balota nigra*	CBS 389.80	JF740247	JF740315	JF740101	_
*L. sydowii*	*Senecio jacobaea*	CBS 385.80	JF740244	JF740313	_	JF740157
*L. veronicae*	Veronica chamaedrys subsp. chamaedryoides	CBS 145.84	JF740254	JF740320	_	JF740160
***Neoleptosphaeria jonesii***	Clematis vitalba	**MFLUCC 16-1442**	**KY211869**	**KY211870**	**KY211871**	**_**
***N. rubefaciens***	***Quercus***	**CBS 223.77**	**JF740243**	**JF740312**	**_**	**_**
*N. rubefaciens*	*Tilia* sp.	CBS 387.80	JF740242	JF740311	_	_
***Ochraceocephala foeniculi***	***Foeniculum vulgare***	**Di3AF1 = CBS 145654**	**MN516753**	**MN516774**	**MN516743**	**MN520147**
*O. foeniculi*	*Foeniculum vulgare*	Di3AF15	MN516766	MN516783	MN516752	_
*Paraleptosphaeria dryadis*	*Dryas octopetala*	CBS 643.86	JF740213	GU301828	_	_
*Pa. macrospora*	*Rumex domesticus*	CBS 114198	JF740238	JF740305	_	_
*Pa. nitschkei*	_	MFLUCC 13-0688	KR025860	KR025864	_	_
***Pa. nitschkei***	***Cirsium spinosissimum***	**CBS 306.51**	**JF740239**	**JF740308**	**_**	**KT389833**
***Pa. orobanches***	***Epifagus virginiana***	**CBS 101638**	**JF740230**	**JF740299**	**_**	**_**
*Pa. praetermissa*	*Rubus idaeus*	CBS 114591	JF740241	JF740310	_	_
***Pa. rubi***	***Rubus*** **sp.**	**MFLUCC 14-0211**	**KT454726**	**KT454718**	**KT454733**	**_**
*Plenodomus agnitus*	*Eupatorium* sp.	CBS 121.89	JF740194	JF740271	_	KY064053
*Pl. agnitus*	*Eupatorium cannabinum*	CBS 126584	JF740195	JF740272	_	_
*Pl. agnitus*	*_*	MFLU 15-0039	KP744459	KP744504	_	_
*Pl. artemisiae*	*_*	KUMCC 18-0151	MK387920	MK387958	MK387928	_
***Pl. artemisiae***	***Artemisia argyi***	**KUMCC 20-0200A**	**MT957062**	**MT957055**	**MT957048**	**_**
*Pl. artemisiae*	*Artemisia argyi*	KUMCC 20-0200B	MT957063	MT957056	MT957049	_
*Pl. biglobosus*	*Brassica rapa*	CBS 119951	JF740198	JF740274	JF740102	KY064054
***Pl. biglobosus***	***Brassica juncea***	**CBS 127249**	**JF740199**	**JF740275**	**_**	**_**
***Pl. changchunensis***	***Poaceae***	**HMJAU 60186**	**OL996123**	**OL897174**	**OL984031**	**OM009247**
***Pl. changchunensis***	***Poaceae***	**HMJAU 60187**	**OL996124**	**OL966928**	**OL984032**	**OL898716**
***Pl. chrysanthemi***	***Chrysanthemum* sp.**	**CBS 539.63**	**JF740253**	**GU238151**	**GU238230**	**KY064055**
*Pl. collinsoniae*	*Vitis coignetiae*	CBS 120227	JF740200	JF740276	_	KY064056
***Pl. collinsoniae***	***_***	**VT02**	**MN653010**	**MN982862**	**MN652269**	**_**
*Pl. collinsoniae*	*_*	KNU-AP100C	LC550566	LC550568	_	_
*Pl. collinsoniae*	*Malus domestica*	KNU-20-A1	LC591836	_	_	LC591846
*Pl. collinsoniae*	*Malus domestica*	KNU-20-A2	LC591837	_	_	LC591847
*Pl. collinsoniae*	*Malus domestica*	KNU-20-A3	LC591838	_	_	LC591848
*Pl. collinsoniae*	*Malus domestica*	KNU-20-A4	LC591839	_	_	LC591849
*Pl. collinsoniae*	*Malus domestica*	KNU-20-C4	LC591840	_	_	LC591850
*Pl. confertus*	*Anacyclus radiatus*	CBS 375.64	AF439459	JF740277	_	KY064057
***Pl. congestus***	***Erigeron canadensis***	**CBS 244.64**	**AF439460**	**JF740278**	**_**	**KY064058**
*Pl. deqinensis*	*_*	CGMCC 3.18221	KY064027	KY064031	_	KY064052
***Pl. dezfulensis***	Brassica napus	**IRAN 4159C = SCUA-Ahm-S41**	**MZ048609**	**_**	**_**	**MZ043102**
*Pl. dezfulensis*	Brassica napus	SCUA-Ahm-S41-2	MZ048610	_	_	MZ043103
***Pl. enteroleucus***	***Catalpa bignonioides***	**CBS 142.84**	**JF740214**	**JF740287**	**_**	**KT266266**
*Pl. enteroleucus*	*Triticum aestivum*	CBS 831.84	JF740215	JF740288	_	KT266270
*Pl. enteroleucus*	*Fraxinus angustifolia*	F-146,176	MN910295	MN910294	_	_
*Pl. enteroleucus*	*Citrus* sp.	ICMP:10937	KT309810	KT309635	_	KT309399
*Pl. fallaciosus*	*Satureja montana*	CBS 414.62	JF740222	JF740292	_	_
***Pl. guttulatus***	**_**	**MFLU 151876**	**KT454721**	**KT454713**	**KT454729**	**_**
*Pl. hendersoniae*	*Pyrus malus*	CBS 139.78	JF740226	JF740296	_	_
*Pl. hendersoniae*	*Salix cinerea*	CBS 113702	JF740225	JF740295	_	KT266271
*Pl. hendersoniae*	*Salix appendiculata*	LTO	MF795790	_	_	_
***Pl. influorescens***	***Fraxinus excelsior***	**CBS 143.84**	**JF740228**	**JF740297**	**_**	**KT266267**
*Pl. influorescens*	*Lilium* sp.	PD 73/1382	JF740229	JF740298	_	KT266273
*Pl. libanotidis*	*Seseli libanotis*	CBS 113795	JF740231	JF740300	_	KY064059
***Pl. lijiangensis***	**_**	**KUMCC 18-0186**	**MK387921**	**MK387959**	**MK387929**	**_**
***Pl. lindquistii***	***Helianthus annuus***	**CBS 381.67**	**JF740233**	**JF740302**	**_**	**_**
*Pl. lindquistii*	*Helianthus annuus*	CBS 386.80	JF740232	JF740301	_	_
*Pl. lindquistii*	*Helianthus annuus*	MF-Ha16-005	MK495988	_	_	MK501790
*Pl. lingam*	_	AFTOL-ID 277	KT225526	DQ470946	DQ470993	_
***Pl. lingam***	***Brassica oleracea***	**CBS 260.94**	**JF740235**	**JF740307**	**_**	**MZ073915**
*Pl. lingam*	*Brassica* sp.	CBS 275.63	MW810266	JF740306	_	MZ073916
*Pl. lingam*	_	CBS 147.24	MW810259	JX681097	_	MZ073914
***Pl. lupini***	***Lupinus mutabilis***	**CBS 248.92**	**JF740236**	**JF740303**	**_**	**KY064061**
***Pl. pimpinellae***	***Pimpenella anisum***	**CBS 101637**	**JF740240**	**JF740309**	**_**	**KY064062**
***Pl. salviae***	Salvia glutinosa	**MFLUCC: 13-0219**	**KT454725**	**KT454717**	**KT454732**	**_**
*Pl. sinensis*	*Plukenetia* sp.	MFLUCC 17-0757	MF072722	MF072718	MF072720	_
***Pl. sinensis***	***Tamarindus* sp.**	**MFLUCC 17-0767**	**MF072721**	**MF072717**	**MF072719**	**_**
*Pl. sinensis*	_	KNU-GW1901	LC550567	LC550569	LC550570	_
*Pl. sinensis*	*Ageratina adenophora*	KUMCC 20-0204	MT957064	MT957057	MT957050	_
*Pl. sinensis*	_	KUMCC 18-0153	MK387922	MK387960	MK387930	_
*Pl. sinensis*	_	KUMCC 18-0152	MK387923	MK387961	MK387931	_
*Pl. sinensis*	_	KUN-HKAS 102227	MK387924	MK387962	MK387932	_
*Pl. tracheiphilus*	*Citrus limonia*	CBS 551.93	JF740249	JF740317	JF740104	MZ073918
***Pl. tracheiphilus***	***Citrus aurantium***	**CBS 127250**	**JF740250**	**JF740318**	**_**	**MZ073919**
*Pl. tracheiphilus*	*Citrus limon*	MUCL 38481	MW810293	MW715037	_	MZ073920
*Pl. tracheiphilus*	*Citrus* sp.	ATCC 26007	MZ049614	MW959165	_	MZ073908
***Pl. triseptatus***	***Daucus carota***	**MFLUCC 17-1345**	**MN648452**	**MN648451**	**_**	**_**
***Pl. visci***	***Viscum album***	**CBS 122783**	**JF740256**	**EU754195**	**EU754096**	**KY064063**
*Pl. visci*	*Viscum album*	CPC:35316	MT223832	MT223924	_	_
*Pl. visci*	*Viscum album*	CPC:35315	MT223831	MT223923	_	_
*Pl. visci*	*Viscum album*	CPC:35314	MT223830	MT223922	_	_
*Pl. wasabiae*	*Eutrema wasabi*	CBS 120119	JF740257	JF740323	_	KT266272
*Pl. wasabiae*	*Eutrema japonicum*	CBS 120120	JF740258	JF740324	_	_
***Praeclarispora artemisiae***	***Artemisia argyi***	**KUMCC 20-0201A**	**MT957060**	**MT957053**	**MT957046**	
*Pr. artemisiae*	*Artemisia argyi*	KUMCC 20-0201B	MT957061	MT957054	MT957047	
***Pseudoleptosphaeria etheridgei***	***Populus tremuloides***	**CBS 125980**	**JF740221**	**JF740291**	**_**	**_**
*pyrenochaeta pinicola*	*Pinus* sp.	CBS 137997	KJ869152	KJ869209	_	KJ869249
*Querciphoma carteri*	*Quercus robur*	CBS 105.91	KF251209	GQ387594	GQ387533	KF252700
*Q. carteri*	*Quercus* sp.	CBS 101633	KF251210	GQ387593	GQ387532	KF252701
*Schleroplectenchymyces clematidis*	*Clematis vitalba*	MFLUCC 17-2180	MT310605	MT214558	MT226675	_
*Shiraia bambusicola*	*Phyllostachys* sp.	GZAAS2 0703	GQ845412	KC460981	_	_
*Sh. bambusicola*	*Pleioblastus* sp.	GZAAS2 0629	GQ845415	KC460980	_	_
*Sphaerellopsis filum*	_	CBS 234.51	KP170655	KP170723	_	KP170704
*Sp. macroconidialis*	*Dianthus caryophyllus*	CBS 233.51	KP170658	KP170726	_	KP170707
***Sp. macroconidialis***	***Allium schoenoprasum***	**CBS 658.78**	**KP170659**	**KP170727**	**_**	**KP170708**
***Sp. paraphysata***	*Cenchrus* sp.	**CPC 21841**	**KP170662**	**KP170729**	**_**	**KP170710**
*Subplenodomus apiicola*	Apium graveolens var. rapaceum	CBS 285.72	JF740196	GU238040	GU238211	_
*Su. drobnjacensis*	*Eustoma exaltatum*	CBS 269.92	JF740211	JF740285	JF740100	_
*Su. drobnjacensis*	Gentiana sp.	CBS 270.92	JF740212	JF740286	_	_
***Su. galicola***	***Galium*** sp.	**MFLU 15-1368**	**KY554204**	**KY554199**	**_**	**_**
*Su. valerianae*	*Valeriana officinalis*	CBS 499.91	JF740252	JF740319	_	_
*Su. valerianae*	*Valeriana phu*	CBS 630.68	JF740251	GU238150	GU238229	_
*Su. violicola*	*Viola tricolor*	CBS 306.68	FJ427083	GU238156	GU238231	KT389849
***Tzeanania taiwanensis***	***Ophiocordyceps macroacicularis***	**NTUCC 17-005**	**MH461123**	**MH461120**	**MH461126**	**MH461132**
*T. taiwanensis*	*Ophiocordyceps macroacicularis*	NTUCC 17-006	MH461124	MH461121	MH461127	MH461133

## Data Availability

All sequences generated in this study were submitted to GenBank. The accession number for the *rpb*2 gene for the new taxon *Plenodomus changchunensis* (HMJAU 60187) is OL944508.

## Supplementary Materials

The following are available online at https://www.mdpi.com/article/10.3390/jof8050416/s1, Figure S1: Phylogram generated from Bayesian inference analysis based on combined ITS, LSU, SSU, and *tub*2 sequence data. Figure S2: Phylogram generated from maximum likelihood analysis based on combined ITS, LSU, SSU, *tub*2, and *rpb*2 sequence data. Figure S3: Phylogram generated from maximum likelihood analysis based on combined ITS, LSU, SSU, and *rpb*2 sequence data. Figure S4: Phylogram generated from maximum likelihood analysis using *rpb*2 sequence data. Figure S5: Phylogram generated from maximum likelihood analysis using *tub*2 sequence data.
