# Supplementary material for: Synopsis of Leptosphaeriaceae and Introduction of Three New Taxa and One New Record from China
Source: J Fungi (Basel). 2022 Apr 19;8(5):416. doi: 10.3390/jof8050416 (PMC9146790; doi:10.3390/jof8050416)
Supplement: Supplementary file 1 [file jof-08-00416-s001.zip › jof-1664670-supplementary.pdf]

SUPPLEMENTARY MATERIAL FOR

Synopsis of *Leptosphaeriaceae* and Introduction of Four New Taxa from China

Rong Xu [1,2], Wenxin Su [1,2], Shangqing Tian [1,2], Chitrabhanu S. Bhunjun [3,4], Saowaluck Tibpromma [5], Kevin D. Hyde [3,6,7], Yu Li [1,*], Chayanard Phukhamsakda [1,2,7,*]

1 Internationally Cooperative Research Center of China for New Germplasm Breeding of Edible Mushroom, Jilin Agricultural University, Changchun 130118, China; xurong@jlau.edu.cn (R.X.); wenxinsu@yahoo.com (W.X.S.); 13689830727@163.com (S.Q.T.)

2 College of Plant Protection, Jilin Agricultural University, Changchun 130118, P.R. China

3 Center of Excellence in Fungal Research, Mae Fah Luang University, Chiang Rai 57100, Thailand; avnishbhunjun@gmail.com (C.S.B.); kdhyde3@gmail.com (K.D.H)

4 School of Science, Mae Fah Luang University, Chiang Rai 57100, Thailand

5 The Center for Yunnan Plateau Biological Resources Protection and Utilization, College of Biological Resource and Food Engineering, Qujing Normal University, Qujing, Yunnan 655011, China saowaluckfai@gmail.com (S.T.)

6 China Innovative Institute for Plant Health, Zhongkai University of Agriculture and Engineering, Guangzhou 510225, P.R. China

7 Jiaxing Key Laboratory for New Germplasm Breeding of Economic Mycology, Jiaxing, 314000, P.R. China

* Correspondence: yuli966@126.com (Y.L.); chayanard91@gmail.com (C.P.)

LIST OF SUPPORTING INFORMATION

Figure S1. Phylogram generated from Bayesian inference analysis based on combined ITS, LSU, SSU and *tub*2 sequence data.

Figure S2. Phylogram generated from maximum likelihood analysis based on combined ITS, LSU, SSU, *tub*2, and *rpb*2 sequence data.

Figure S3. Phylogram generated from maximum likelihood analysis based on combined ITS, LSU, SSU and *rpb*2 sequence data.

Figure S4: Phylogram generated from maximum likelihood analysis using *rpb*2 sequence data.

Figure S5: Phylogram generated from maximum likelihood analysis using *tub*2 sequence data.

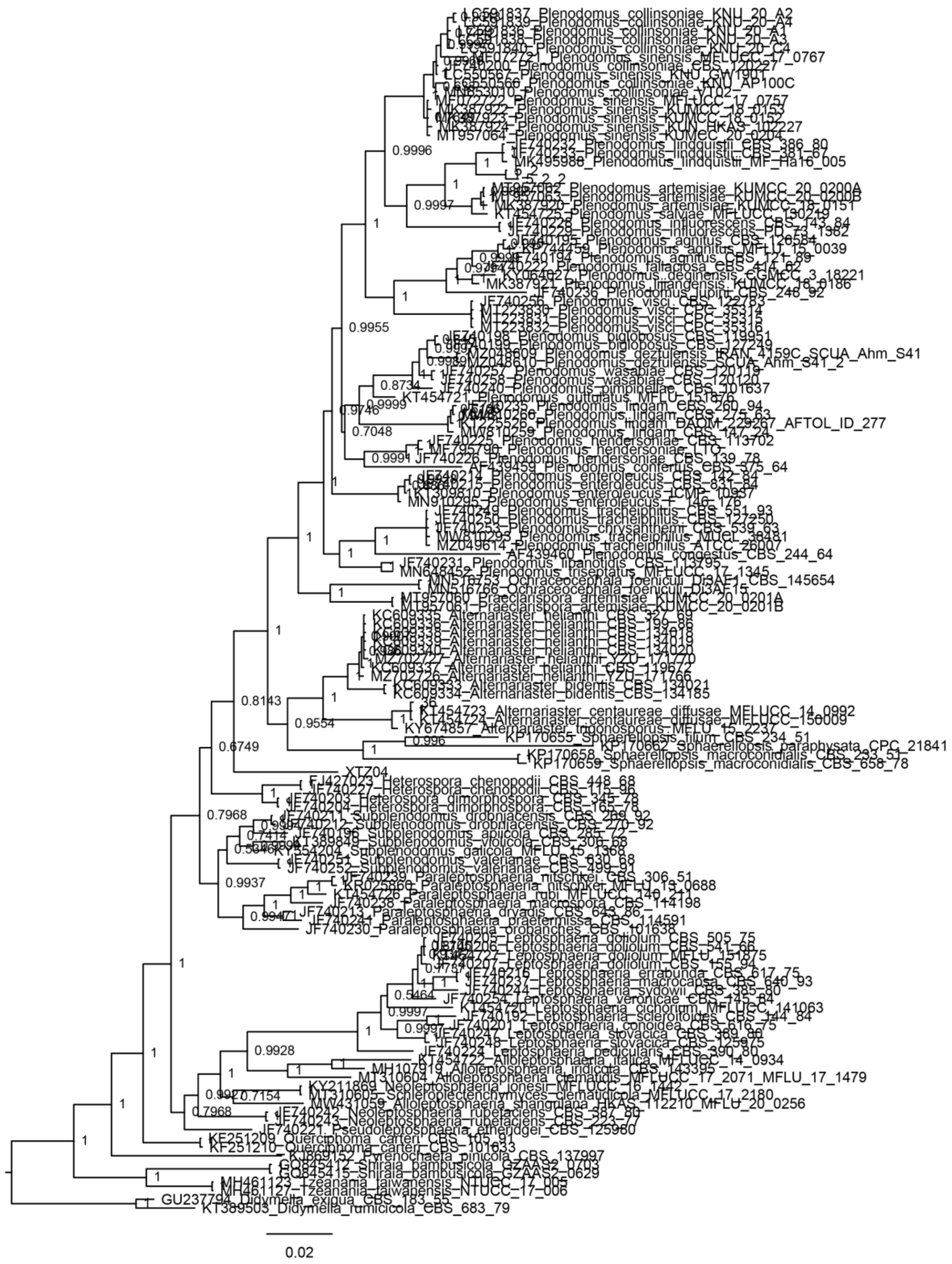

Figure S1. Phylogram generated from Bayesian inference analysis based on combined ITS, LSU, SSU and *tub2* sequence data. The strains are named following the accession number of the ITS region.

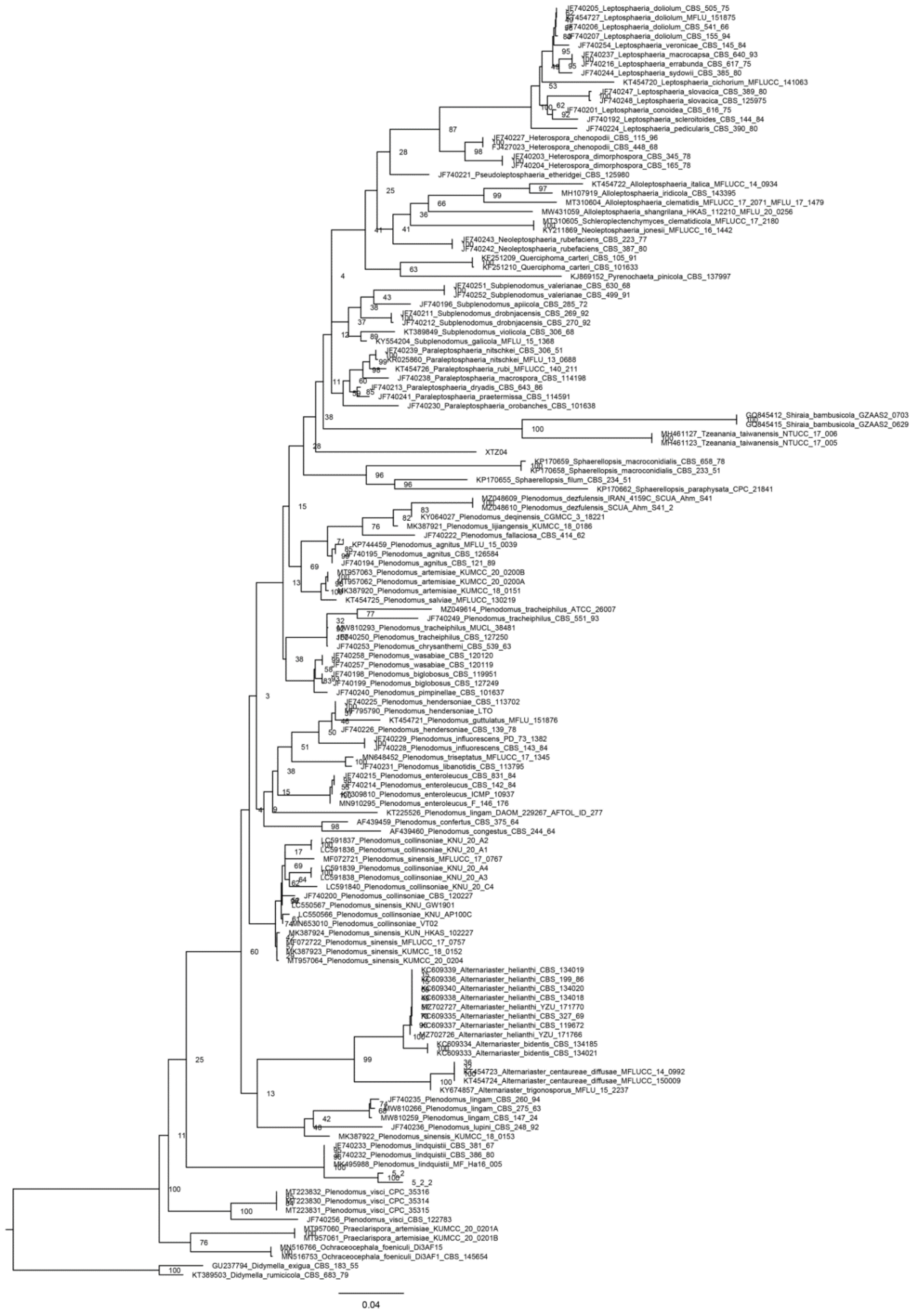

Figure S2. Phylogram generated from maximum likelihood analysis based on combined ITS, LSU, SSU, *tub*2, and *rpb*2 sequence data. The strains are named following the accession number of the ITS region.

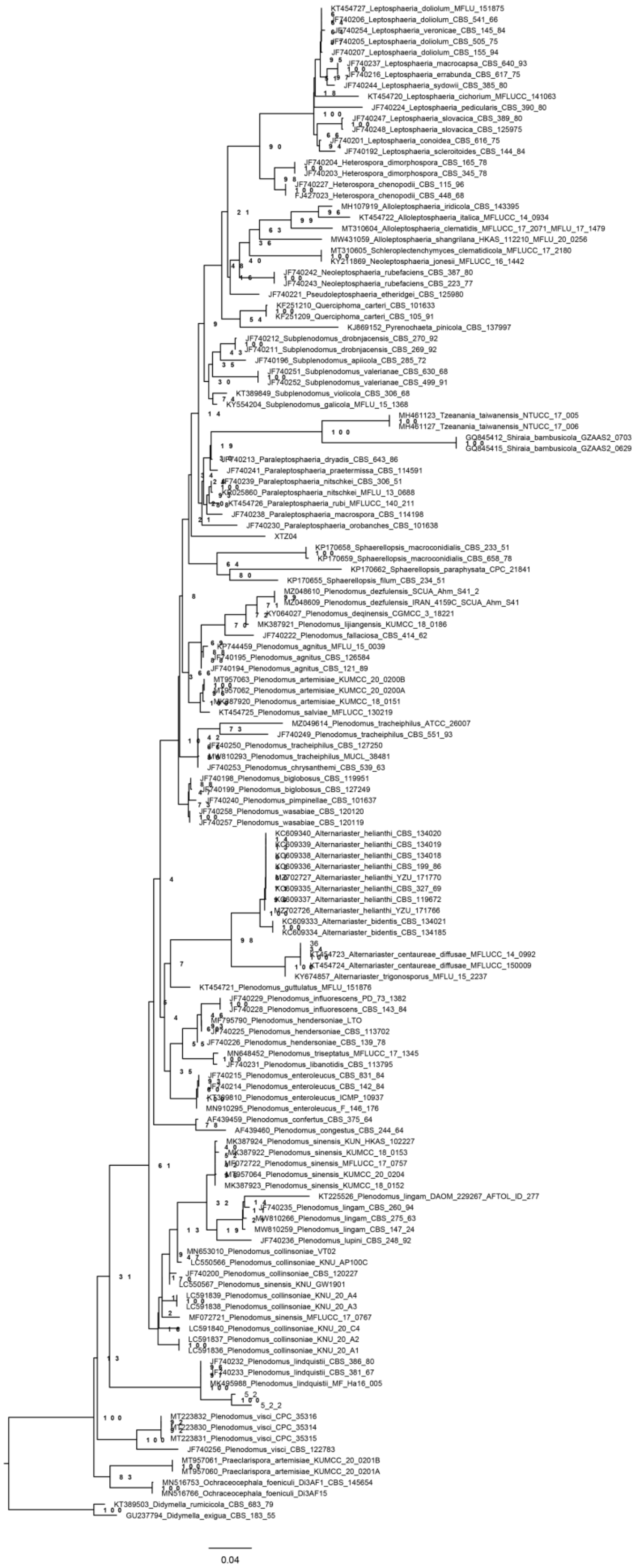

Figure S3. Phylogram generated from maximum likelihood analysis based on combined ITS, LSU, SSU and *rpb*2 sequence data. The strains are named following the accession number of the ITS region.

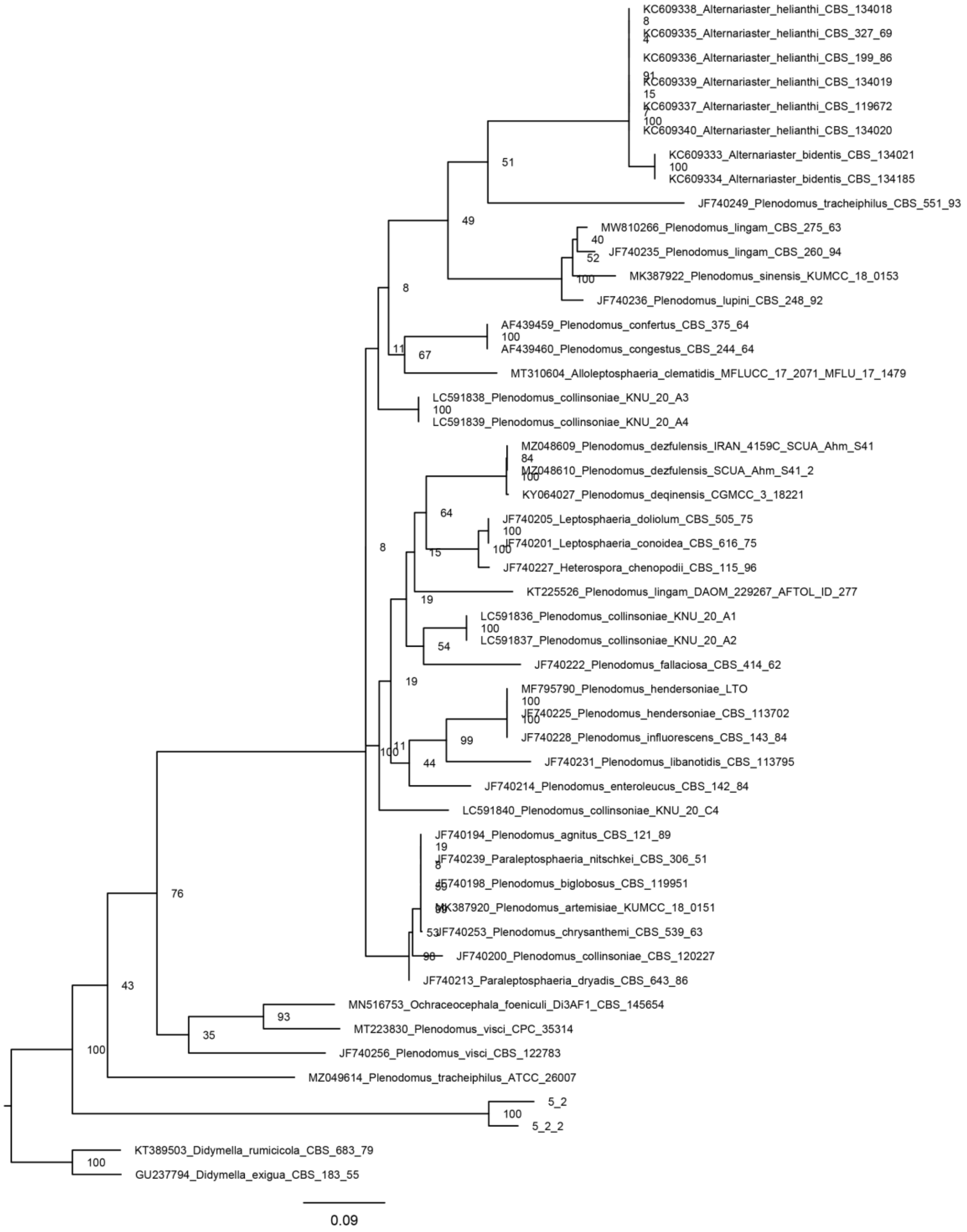

Figure S4: Phylogram generated from maximum likelihood analysis using *rpb*2 sequence data. The strains are named following the accession number of the ITS region.

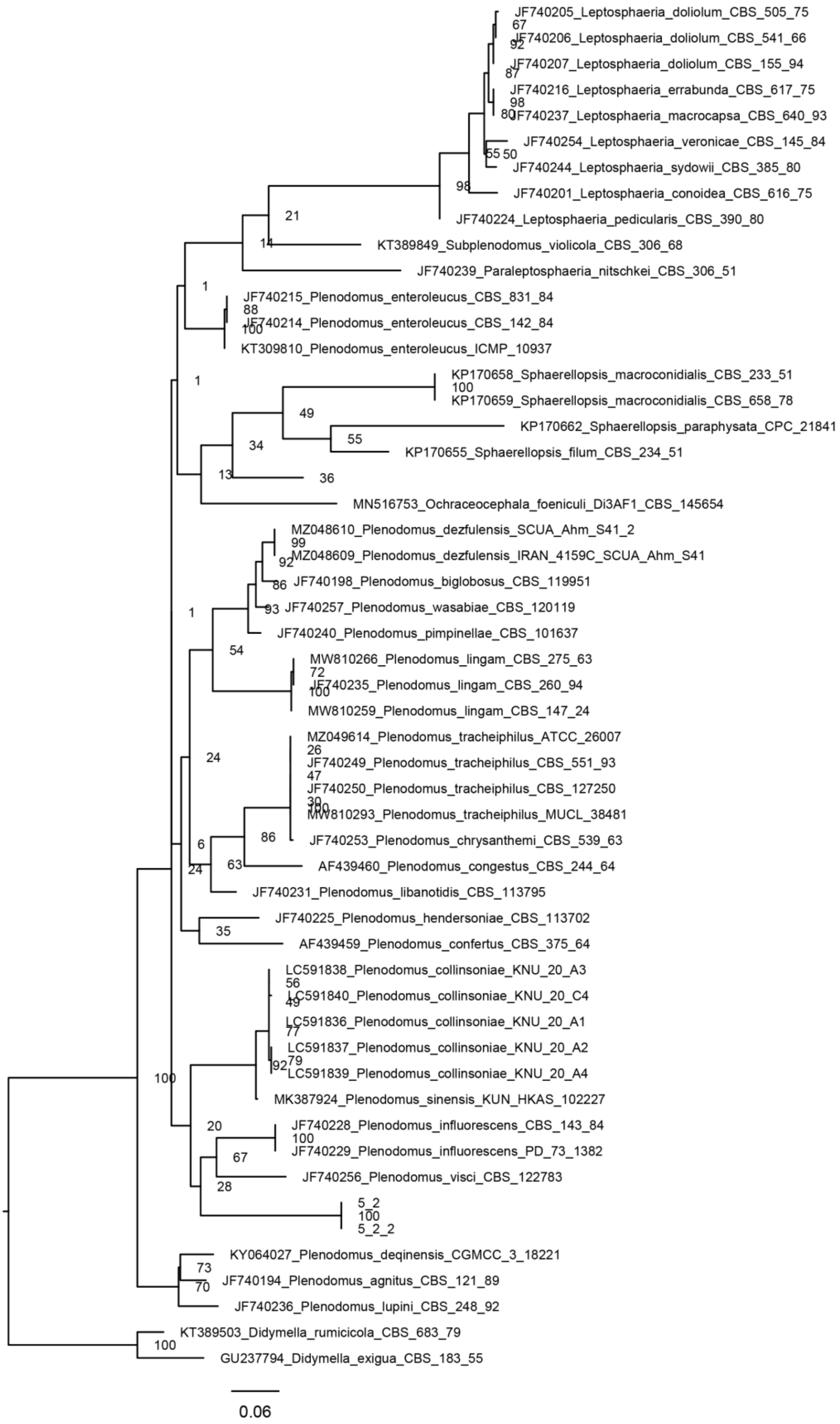

Figure S5: Phylogram generated from maximum likelihood analysis using *tub*2 sequence data. The strains are named following the accession number of the ITS region.
